# Electromagnetic Energy Harvester Targeting Wearable and Biomedical Applications

**DOI:** 10.3390/s24072311

**Published:** 2024-04-05

**Authors:** Gabriel Digregorio, Jean-Michel Redouté

**Affiliations:** Department of Electrical Engineering, ULiège University, 4000 Liège, Belgium; jean-michel.redoute@uliege.be

**Keywords:** energy harvesting, characterization, inertial electromagnetic energy harvesters

## Abstract

This work presents a miniaturized electromagnetic energy harvester (EMEH) based on two coils moving in a head-to-head permanent magnet tower. The two coils are separated by a set distance so that the applied force moves the EMEH from one equilibrium position to another. In this configuration, the harvester produces energy in two different working modes: when a force is applied to the moving part or when an external random acceleration is applied to the whole system. A custom test bench has been designed to characterize the behavior of this energy harvester under a variety of conditions encountered in wearable applications. Notably, at 10 Hz and 1.32 g RMS acceleration, our inertial EMEH demonstrates its capability to sustain a consistent output power of 1696 μW within a total volume of 22.39 cm^3^, showcasing its efficiency in environments with erratic stimuli typical of wearable and biomedical applications. The presented EMEH is compared with reported inertial EMEH structures to extract its design limitations as well as future improvements, situating the present work in a comprehensive state-of-the-art and defining a generic performance target for biomedical and wearable applications.

## 1. Introduction

Energy Harvesters (EHs) often refer to small-scale devices capable of converting wasted energy from ambient sources into electrical energy or chemical energy. The latter are then used to power electronic circuits such as sensors for ambient monitoring or wireless sensor nodes [1]. The major interest in harvesting energy lies in the design of autonomous miniaturized electronic circuits and sensing platforms that minimize battery replacement or completely replace the need for batteries in low-power devices. Among miniaturized energy harvesters, the technology based on electromagnetic energy scavenging is retaining its popularity [2,3], especially for embedded (“buried”) electronics where the finite lifespan of batteries is a leading problem [4]. Electromagnetic Energy Harvesters (EMEHs) benefit from a higher mechanical to electrical energy conversion efficiency compared to EHs based on electrostatic and piezoelectric technologies when the available volume is larger than a few cubic centimeters and the mechanical movement amplitude is large [5,6]. Also, their close relationship with reported power generators makes them the appropriate technology when sufficient space is available. In summary, this technology is suitable for harvesting an adequate amount of energy and producing a few tens of μW for a volume in the range of a few cubic centimeters [7].

**Figure 1:**
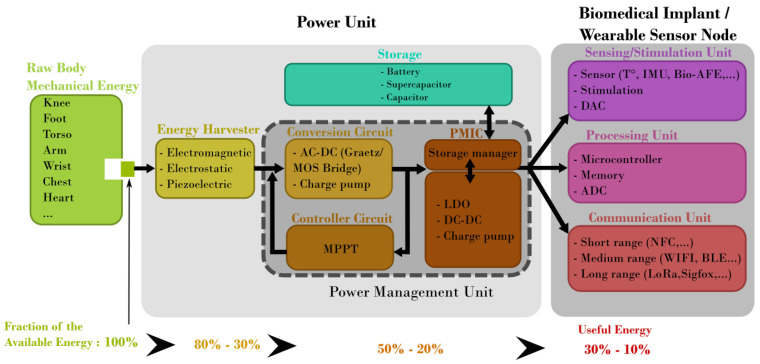
Energy transfer diagram for motion-based harvesters used in wearable and biomedical applications.

Human energy expenditure comes from different body activities and the resulting available raw energy can easily reach a few hundreds of μW [8]. Furthermore, when the available space is larger than a few cm^3^, EMEHs are a suitable technology which can easily benefit from large movement amplitudes coming from the motion of arms and legs, or large forces and momentum stemming from the movement of large joints, such as the knees and heels. Figure 1 shows the main building blocks that compose a generic Wireless Sensor Node (WSN) powered by an energy harvesting system. Generally speaking, most of the energy extraction limitation takes place during the conversion of the raw mechanical energy to electrical energy [8,9]. However, it is often preferable for wearable and biomedical applications to extract a fraction of the available energy in order to perturb as little as possible the natural movement without causing any disruption, discomfort or harm to the subject. From this point of view, it is considered that 100% of the mechanical energy corresponds to what can effectively be taken from the body movement without impairment to the subject. Losses are therefore taken into account from there. Regarding the power target, the required energy depends strongly on the application. Indeed, for regulating heart activity (pacemaker) a few μW are required [10] while for cochlear implants, a few mW are required [11]. In case wireless data transmission is required (e.g., in Wireless Sensor Networks), the energy consumption is mainly used by the communication unit [1].

In most biomedical applications, the sensor, the microcontroller and/or the wireless system can be in standby mode most of the time in which a tiny amount of energy (a few tens of μW) is required. Additionally, the volume of transmitted data is often limited and time-spaced in most healthcare monitoring devices and wireless body sensor networks. The required average power for such a device is therefore intrinsically related to the applicable scenario (sleeping mode/active mode time ratio, measurement frequency, transmission data rate and regularity, *…*). In order to extract the power target that an energy harvester must achieve to power a biomedical device, three scenarios are proposed in Table 1 to identify orders of magnitude for the required energy.

These performance targets are not arbitrary but have been carefully chosen to ensure that the EMEHs are suitable for their corresponding intended applications. They are derived from a compilation of several studies that define energy or power thresholds at which the respective energy harvesters become relevant. For instance, Dogan et al. [16] provide detailed insights into the power consumption of an ultra-low-power multi-core architecture designed for wearable health monitoring systems, which was found to be around 0.85 to 1.11 mW for various workloads. On the other hand, the study Shi et al. [17] elaborates on the harvested power output from human walking, often between milliwatts and microwatts. In the context of biomedical and wearable applications, these targets are particularly relevant as they directly impact the functionality and efficiency of the devices. These applications often involve embedded systems where access is difficult, constrained, or too costly, making the optimization of electromagnetic energy harvesters (EMEHs) crucial. Therefore, understanding and defining these performance targets is a critical aspect of this work, as it allows to design and optimize EMEHs that are not only efficient but also tailored to the specific needs of the biomedical and wearable applications they are intended for.

An average energy consumption of 53.45 mJ can be considered for transmitting and receiving 1 Mb of information through the Bluetooth Low Energy 5.0 (BLE) transmission technology at 121 kbps [18]. The power consumption during continuous operation of a sensor platform with microcontroller acquisition at least an order of magnitude smaller. As a baseline, it is safer to consider a generic target of 100 μW to be able to power a sensor, the acquisition electronic system, the microcontroller, and the transmission system. This target is not an absolute constraint for biomedical and wearable applications but serves as a reasonable starting point to evaluate when an energy harvesting solution becomes attractive compared to a battery.

Our research introduces an advanced electromagnetic energy harvester optimized for wearable and biomedical applications. This system adeptly enhances power density through innovative mechanisms and design optimizations, ensuring maximal energy capture in minimal volume. By expanding operational bandwidth, it accommodates a wide range of human movements, from subtle to dynamic, ensuring efficient energy conversion under varied conditions. Additionally, it guarantees reliable output power in the face of erratic and unpredictable human motions, employing adaptive mechanisms that maintain performance across different activity levels. Together, these advancements address critical barriers in energy harvesting technology, paving the way for more sustainable and effective wearable devices. By leveraging a unique dual-coil and head-to-head magnet configuration, we achieve enhanced efficiency and adaptability to erratic stimuli movements, setting our design apart from existing solutions. This device offers a versatile approach to energy generation from human motion, underscoring its potential to advance the development of self-powered wearable technologies.

This paper is structured as follows. First, a state-of-the-art comparison of various EMEHs and reporting their performances is presented in Section 2. This review situates the performances and the behavior of the proposed miniaturized EMEH. The design of the presented EMEH is detailed in Section 3. This harvester is composed of two central ferromagnetic cores wound with a thin copper wire. An optimization process aimed at reducing the resulting vertical force needed to switch the EH from one equilibrium position to another has been reported in [19]. The test bench is described in Section 4. Finally, the experimental results from the proposed EMEH are discussed and compared with respect to the state of the art in Section 5.

## 2. State of the Art Comparison of EMEHs

Various electromagnetic energy harvesters (EMEHs) employ distinct approaches to optimize the output electrical power while minimizing the size of the harvester. This profusion of EMEH topologies, grounded in diverse mechanisms and designs, presents significant challenges in achieving an objective and fair comparison across proposed architectures. Acknowledging these complexities, our study carefully selects EMEHs for comparison based on the comprehensiveness and relevance of their reported data, particularly those pertinent to wearable and biomedical applications and where the volume of the proposed prototypes is coherent with the intended application. By doing so, we aim to navigate the inherent challenges posed by the wide variability in experimental setups and the lack of uniform characterization parameters, which could otherwise impede a straightforward analysis. This selective approach facilitates a balanced and informative comparison, concentrating on designs that offer meaningful insights into performance metrics critical for assessing the utility of EMEHs in their intended applications. Our analysis is designed to highlight both achievements and areas for potential improvement within the field, providing an overview rather than an exhaustive meta-analysis of all miniaturized inertial EMEHs.

Also, those harvesters are designed according to specific hypotheses related to a specific application which exacerbates their bench-marking. Indeed, often the weight-based and volume-based power density is given but these parameters depend on the type of external input excitation and the selected operating point which are directly related to the input excitation. It is therefore preferred to compare the energy harvesters with respect to the normalized power density to take into account the volume in combination with the corresponding input excitation [20]. Furthermore, to ease the comparison with batteries and the target power and volume established in this work, the powers of inertial EMEHs have been compared to the energy density (per unit volume). One major drawback is that the source of energy is often considered to be unlimited which is a good approximation in applications where the harvester does not influence the raw energy source. This approach is biased as it artificially boosts the generated output power by considering applications where the harvester does not affect the input source excitation. The energy conversion efficiency that corresponds to the ratio between the measured electrical power and mechanical power (ability of the harvester to use the raw energy source as efficiently as possible without wasting the input energy) is therefore complicated to compare and often not reported. Also, this parameter depends on various testing conditions, especially the used load and power management unit (PMU) to condition and temporarily storing the energy. For these reasons, the efficiency is not a privileged criterion for comparing EHs.

A thorough investigation has been performed on a set of representative designs in order to compare these on an equal footing. Only miniaturized inertial harvesters using electromagnetic transduction have been selected. All designs have been tested with an external sinusoidal excitation, and the harvesters which were tested in real conditions where the external excitation can’t be approximated by a sinusoidal excitation source, were excluded.

**Figure 2:**
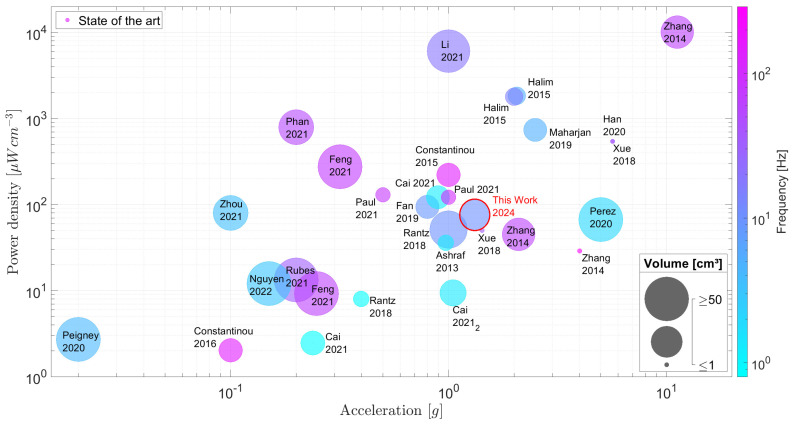
Comparison chart gathering on the same footage various inertial EMEH experimentally tested in the literature [3,23,24,25,26,27,28,29,30,31,32,33,34,35,36,37,38,39,40,41,42]. The power densities, accelerations, frequencies of operation and volumes of EMEHs are depicted on the same figure in order to highlight the dependencies related to those parameters. The red circle identifies the proposed EH discussed in this work.

Figure 2 compares the selected inertial EMEH designs proposed in the literature. Three variables have been selected. The first one is the applied acceleration expressed in *g* (where 1 g =9.81 m/s^2^) which defines a convenient measure of the strength of the input excitation signal. Secondly, most of the results highlighted by the authors correspond to the respective harvester working at its resonant frequency. This approach can be debated when considering biomedical and wearable applications where the harvester is subjected to erratic and random stimuli and to a range of low frequencies far from its resonant and optimum frequency. Therefore, the optimum working frequency is depicted with the color scale where the harvester produces its maximum power. The third parameter is the power density in μW/cm^3^ which normalizes the produced power to the volume of the harvester. The volume is preferred to the mass in the field of wearable electronics, as the weight is negligible compared to the volume and whereas the latter can be a source of discomfort and often makes it unpractical to use. The power is, when possible, extracted from the current and voltage measured on a purely restive load connected at the output of the harvester coils. Some harvesters have been characterized with a dedicated PMU in which case the latter values were used. Finally, the total volumes of the proposed harvesters are depicted by the bubble size in Figure 2. A log scale is used to sketch the volume of each EH where the limit range has been set to [1; 100] cm^3^. Therefore, EH with a total volume equal to or bigger than 100 cm^3^ are plotted with the same bubble size. On the other hand, volumes equal to or smaller than 1 cm^3^ are also plotted with the same bubble size. The harvested electromagnetic energy evolves as a function of the cube of the dimensions of a power generator [21]. The active volume [22] is often taken as the reference volume to compute all the resulting quantities such as the normalized power density. In this work, we have approximated the total volume of each harvester by considering an equivalent minimal volume capable of encapsulating the entire system. In that way, all the considered harvesters are benchmarked with common parameters by considering their performance and power density in a real situation.

Due to the variety of proposed EMEH designs and the diversity of experimental setups to test each EH, Figure 2 extracts the general tendencies and the common behavior without discriminating quantitatively the selected EHs. As can be observed, the proposed EMEH (Section 3 fits the imposed target for wearable and biomedical applications in terms of size, working frequencies, accelerations, and output power. One can observe in Figure 2 an increase in power density as the applied acceleration gets larger. Also, the figure highlights the wide range of power densities for the selected EMEH which leads to difficulties to predict the output power density for a given volume.

A volume-based figure of merit FoMv is a suitable indicator where each EMEH is compared with a cubic-shaped EMEH of equivalent volume with the proof-mass occupying half of the volume and the remaining space is reserved for its displacement [43]. This reference is chosen as the theoretical maximum of the extracted power for a given volume *V*, frequency ω/2π, and imposed amplitude displacement $Y_{0}$. Gold is taken as a reference material for the proof mass as its density can be considered the highest in MEMS and miniaturized electronics. The FoMv translates the discrepancy of a given inertial EMEH to its idealized form:

(1) $$\mathrm{FoMv}=\frac{\mathrm{Useful}\;\mathrm{Power}\;\mathrm{Output}}{\frac{1}{16}Y_{0}\rho_{\mathrm{Au}}V^{\frac{4}{3}}\omega^{3}}.$$

**Figure 3:**
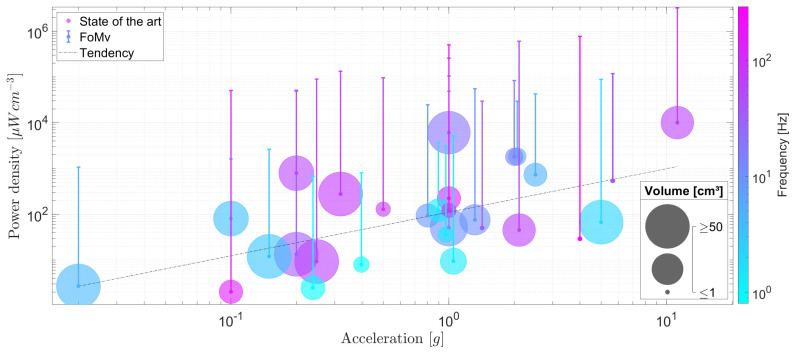
Performances of selected inertial EMEH and the distance from their idealized power densities (based on FoMv). The power densities, accelerations, frequencies of operation, and volumes of EMEHs are depicted in the same figure in order to highlight the dependencies related to those parameters. Their attached bar represents the distance from their idealized yield.

This figure of merit corresponds to a maximum output power generated by an EMEH as it does not take into account the space needed for the frame the coils and the spring system. Also, it assumes that the damping is completely related to the harvested energy in a way that no energy is lost. For the above-mentioned reasons, practical implementations of EMEHs stay way below this upper limit given for an idealized EMEH, as shown in Figure 3. Based on these observations, one can consider that the present state-of-the-art EMEHs stay below 10% of their corresponding ideal output power with an average at 1.03%. Lastly, one should consider the limited load factor of EMEHs in the field of wearable devices and biomedical applications compared to batteries. EMEHs produce energy either when external forces or random vibrations are applied onto the system, i.e., when a person moves, walks or exerts pressure on the EMEH. Depending on the origin of the raw energy listed in Figure 1, energy is produced during a small fraction of the daytime. This has the effect of reducing the load factor (percentage of time in a day where the harvester produces energy). Depending on the application, batteries may then, for a given application, become necessary and therefore represent a major competitor to EMEHs (refer to Section 5).

## 3. Design of the Proposed EMEH

**Figure 4:**
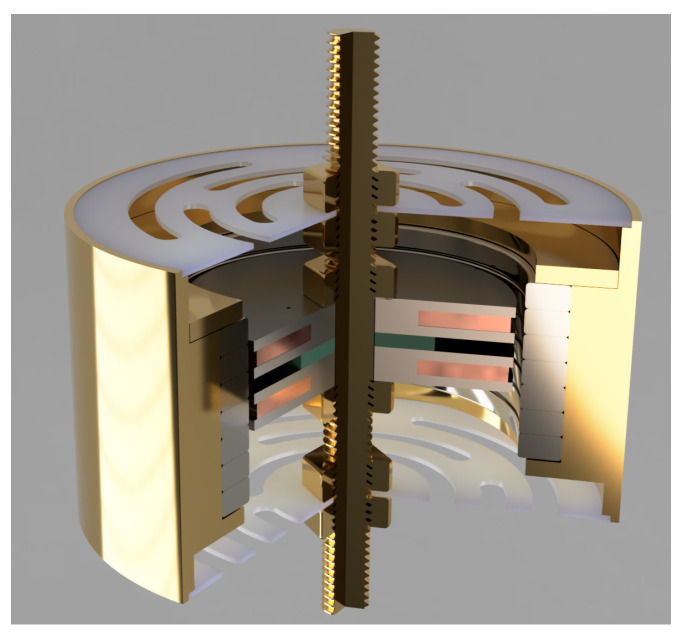
3D rendering of the proposed EMEH.

**Figure 5:**
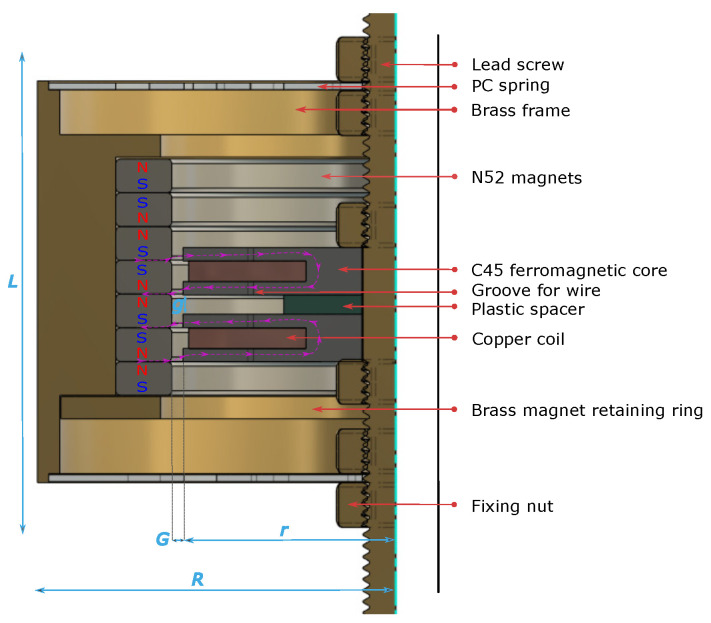
Cross section of the miniaturized EH architecture (dimensions are in mm: only the left half-plane is shown as the EH is symmetrical around its axis).

The proposed EMEH’s geometry is shown in Figure 4 and Figure 5. An analytical and numerical analysis has been reported in [19]: this work focuses on its experimental electrical and mechanical characterization. Two coils machined in C45 steel are wound with 100 μm copper wire. The two ferromagnetic cores are separated by a 1.83 mm thick plastic spacer to avoid cross-flux between each coil. This gap is crucial for minimizing the magnetic sticking force between the moving part (permanent magnets) and the stationary part (the dual coil) as it is shown and detailed in Section 5. Thus, the transition from one equilibrium position to another can be facilitated by adjusting the compensation of forces experienced by each coil. By strategically managing this magnetic interaction, the system achieves a delicate balance, enabling smoother motion and enhancing the overall performance of the device. This approach underscores the importance of precise spatial configuration in the design of magnetic systems, as detailed in the findings of the referenced study. N52 1.5 mm thick ring magnets are stacked in a head-to-head configuration to increase the magnetic field gradient along the axial direction. Springs are 3D printed in polycarbonate (PC) with an FDM 3D printer. This material is suitable for low-stiffness springs compared to traditional spring steel while avoiding too much creep compared to the often-used thermoplastic Polylactic Acid (PLA). The spring design has been studied to achieve 175 N/m axial stiffness. This specific stiffness was experimentally chosen to fulfill two objectives. The first is to keep the ferromagnetic core perfectly aligned with the magnet, ensuring the highest radial stiffness. The second is to minimize the force required to traverse all the equilibrium positions, necessitating the lowest axial stiffness. In this type of diaphragm spring, both axial and radial stiffnesses are correlated. A compromise has been made to prevent sticking between the ferromagnetic core and the magnet when the system is subjected to heavy accelerations and stimuli. Finally, the frame is made of brass and machined to fit the diameters of the magnets and the springs. A thin retaining ring made of brass is pushed and crimped at the bottom of the stack of head-to-head magnets. A long screw on which the coils and springs are fixed through nuts is machined in brass and fixes the harvester firmly onto the test bench. The corresponding (total) volume of the harvester, also considering the space needed for the spring displacement, is equal to 22.39 cm^3^. All the parameters and dimensions of the tested EMEHs are listed in Table 2.

Head-to-head magnets were employed in the present design to create a magnetic field with a strong gradient in one direction (along the *z*-axis). Indeed, the electromotive force (e.m.f) induced within a coil is proportional to the variation of the position with respect to time (velocity) and the variation of the magnetic flux density with respect to the position:

(2) $$\begin{array}{cc} {e.m.f}_{coil1} & =-N_{\mathrm{coil}1}\frac{d\phi_{cor1}}{dy}\frac{dy}{dt},\\ {e.m.f}_{coil2} & =-N_{\mathrm{coil}2}\frac{d\phi_{cor2}}{dy}\frac{dy}{dt}, \end{array}$$

where $N_{\mathrm{coil}1}=N_{\mathrm{coil}2}$ is the number of turns in the winding on each coil and $\phi_{cor1}$ = $\phi_{cor2}$ is the average magnetic flux in the central region on both ferromagnetic core. By using this custom double coil structure, the magnetic field can be kept strong while the magnetic force along the *z*-axis can be reduced as already studied and demonstrated in a previous work [19]. The magnetic flux density is effectively captured by the flanges of the ferromagnetic cores when they are facing the interface between the two magnets. The gap between the coils and the magnet tower was analyzed through FEA simulation and was chosen to minimize saturation in the flanges while maximizing the flux variation in each coil. This layout facilitates multiple stable positions during the excitation of a system that uses inertial mass, a strategy that addresses the broad range of excitation frequencies often encountered in energy harvesters.

Furthermore, the spacing between the two cores, and so two pairs of flanges, has been studied to minimize the force required to transition from one equilibrium position to another, thereby facilitating the movement of the ferromagnetic core between different equilibrium positions. This aspect further contributes to the advantages of our head-to-head magnet array design in comparison to previous EMEHs. Finite element analysis has been used to optimize the geometry leading to the design proposed in Figure 5.

By changing the distance between the coils, the stable positions can be tuned and reduced magnetic forces along the *z*-axis can be achieved. Therefore, the proposed EMEH can be tuned to perform throughout different ranges of acceleration frequencies and amplitudes. When the system is accelerated, energy is stored in the spring until it is released, causing the magnets to move and create a pulse of current. This combination of strategies allows for a fast oscillating movement to be triggered through a rapidly changing magnetic field, either by applying an external slow movement to the system (for EHs relying on direct force application) or by applying acceleration to the system (for inertial EHs). This work focuses on the inertial EMEH behavior in order to compare the proposed design to the state of the art.

## 4. Experimental Setup and Custom Test Bench

**Figure 6:**
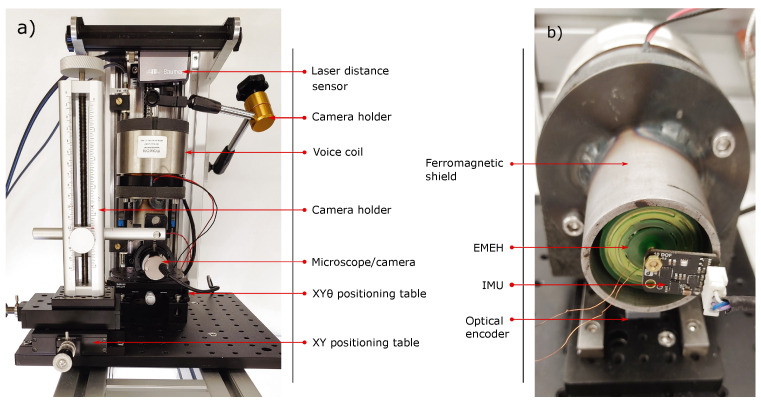
(**a**) Test bench. (**b**) A close-up view of the EMEH fixed to the voice coil.

To test various energy harvesters based on inertial proof masses and direct applied forces for biomedical and wearable applications, a custom test bench capable of reproducing a large variety of mechanical excitations, movements and loads has been designed [19]. The test bench (Figure 6) is composed of a 50 W hollow voice coil actuator (linear motor) able to perform small displacements (25 mm stroke) at relatively high frequencies ([0; 20] Hz for 5 mm amplitude). The moving part (coil) of the voice coil is guided through linear ball bearings and guiding rails and is driven by a servo motor driver with a PID control connected to a 1.25 micron linear optical encoder (quadrature resolution) and is able to reach accelerations up to 3 g with a feedback loop in position to ensure a smooth oscillation and reduce drift and overshoots. The main control is performed by an Infineon TC275 32-bit three-core microcontroller. A compact Inertial Measurement Unit (IMU) sensor has been added to the test bench and is composed of an Analog Devices ADXL345 accelerometer. The unit is firmly fixed to the energy harvester holder and is able to detect short acceleration pulses and shocks in the range of [−16; 16] g and is used to extract the RMS acceleration perceived locally by the tested EMEH. A graphical user interface (GUI) allows conducting different experiments and comparing different harvesters on the same basis. A data acquisition system is synchronized with the test bench in order to perform real-time electrical characterization of the harvester and extract the output power during the applied stimuli). A resistive load of 25Ω is connected to both coils to maximize the output power dissipated in the load. No PMU was used in the experiment to focus on the output power of the harvester and extract its nominal output power under real stimuli.

## 5. Results and Discussion

**Figure 7:**
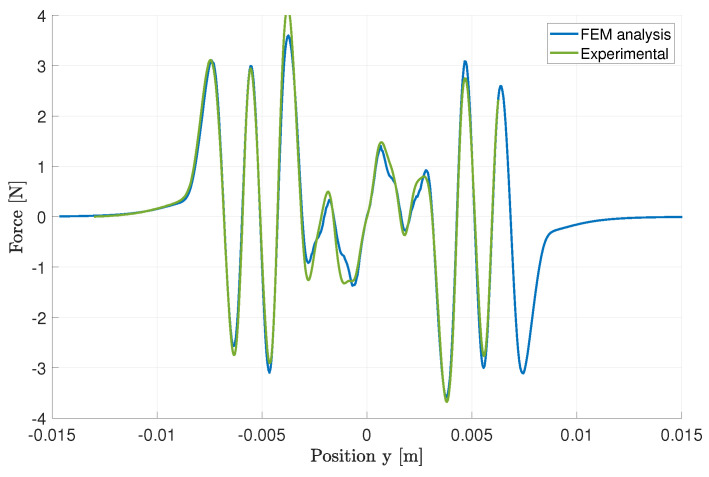
Magnetic force along the vertical axis (z direction) between the head-to-head magnet tower and the ferromagnetic core. This plot shows the finite element analysis (FEA) processed on the measured geometry including the imprecisions due to machining.

First, the magnetic force between the magnet tower and the ferromagnetic cores was measured using the test bench, and compared with a finite element analysis (FEA). The springs were removed and the coil axis was fixed to the load cell (fixed part), while the magnet tower and brass frame were attached to the main carriage (moving part). The camera was used to ensure perfect alignment during the measurements. The resulting vertical force is shown in Figure 7. A very good match between the FEM model and the experimental measurement of the vertical force experienced between the ferromagnetic cores and magnets is observed, notably in the region of interest for displacement allowed between −3 and +3 mm. This alignment highlights the accuracy of our FEA model in predicting the magnetic interaction under the specific conditions tested, reinforcing the validity of our approach for analyzing and designing magnetic systems in this operational range.

**Figure 8:**
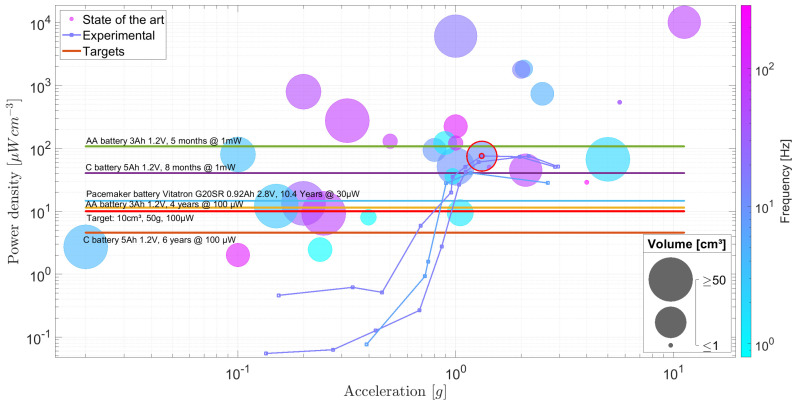
Experimental characterization of the proposed miniaturized inertial EMEH tested at frequencies of 7 Hz and 10 Hz, for a displacement amplitude ranging from 1 mm to 10 mm. This figure also includes performance targets corresponding to select batteries, providing a direct comparison with potential energy storage competitors. Additionally, it encompasses a comprehensive performance overview of other energy harvesters detailed in Figure 2, representing a state-of-the-art benchmark. This integration of experimental results and comparative benchmarks offers a holistic view of the EMEH’s performance relative to existing energy solutions and state-of-the-art technologies.

Figure 8 illustrates the experimental findings of the proposed inertial EMEH for two distinct frequencies. This figure encapsulates a wealth of information, enabling a straightforward comparison of different parameters across various energy harvesters and their direct competitors, namely batteries, which remain unaffected by changes in acceleration or excitation frequency. By presenting multiple data points across a spectrum of accelerations, we aim to provide a clear demonstration of our system’s performance and emphasize the variability in output power per unit volume that miniaturized inertial EMEH systems exhibit in response to differing applied stimuli. This aspect of performance variability, significantly influenced by the specific conditions of acceleration and frequency, is often overlooked in existing literature, which tends to focus on peak performance metrics at singular operational points. Our detailed analysis, as shown in Figure 8, draws clear trends and insights, effectively juxtaposing the dynamic performance of EMEHs against static energy storage solutions. This approach not only sheds light on the inherent performance characteristics of EMEH systems but also underlines the importance of considering real-world application scenarios where variations in acceleration and frequency are commonplace, thus offering a more nuanced and comprehensive understanding of EMEH performance, its potential applications, and limitations.

**Figure 9:**
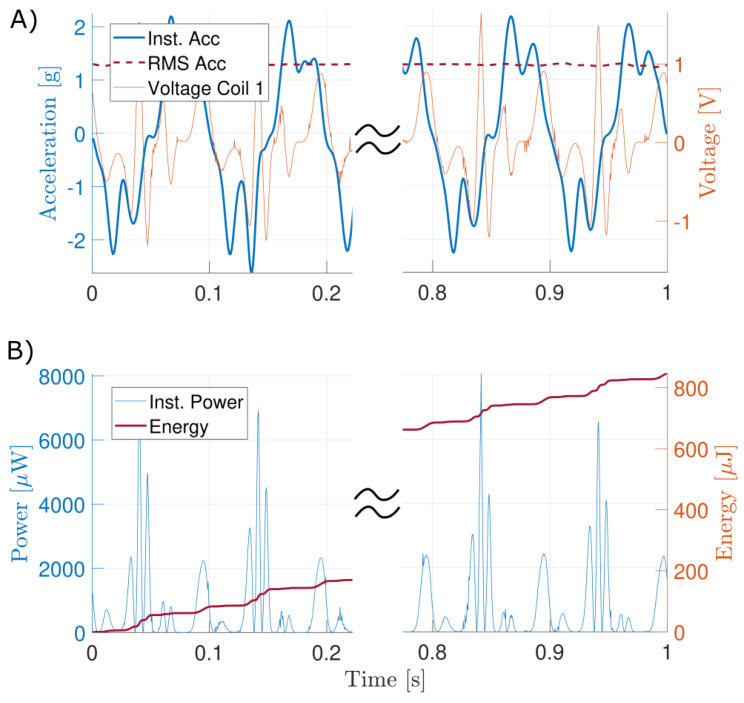
(**A**) Voltage measured across a 25 Ω load connected on a single coil for an external excitation at 10Hz and with an amplitude of 8 mm. (**B**) Power and energy harvested on a single coil by the inertial EMEH.

The proposed EH was tested at two different frequencies: 7 Hz and 10 Hz. Those two frequencies are chosen as they correspond to maximal representative frequencies for wearable and biomedical applications [44]. It should be noted that frequencies of movements experienced by the body during regular activity typically lie below 2 Hz with accelerations less than 2 g [45]. For each frequency, the displacement amplitude was set from 1 mm to 10 mm in order to sweep the range of admissible accelerations. The movement was recorded by the accelerometer fixed on the ferromagnetic shield close to the EMEH. Due to the distance between the linear guiding rails and the EMEH, the experienced oscillation was not perfectly harmonic. Therefore, the RMS acceleration was extracted to compare the performance of the harvester with the state of the art described in Section 2. Figure 9 shows the generated power during 1 s for a 10 Hz and 7 Hz applied sinusoidal acceleration with a displacement of 8mm. The energy accumulated during the oscillation is also indicated alongside the instantaneous power. The same resistive load of 25Ω was used during these tests. A ferromagnetic shield was placed around the tested EMEH in order to reduce the influence of the voice coil on the energy harvester coils (see Figure 6). The impact of this coupling on the harvested energy is negligible as can be seen in Figure 9. Indeed, high-frequency spikes do not contribute to the harvested energy.

An evident limitation of the proposed energy harvesting system is the observable saturation effect, where an increase in acceleration leads to a plateau in the harvested power. This behavior is primarily attributed to the spring system, which plays a crucial role at acceleration levels exceeding 1 g. The design was specifically optimized to achieve peak performance around accelerations of 1 g, as previously discussed. Furthermore, the unique configuration of the double ferromagnetic core and magnet tower creates unstable equilibria, leading to erratic oscillatory behavior. This can result in reduced performance as the oscillation frequency or amplitude increases. Notably, the power curves at 7 Hz and 10 Hz intersect when plotted against acceleration, illustrating the unpredictable nature of such highly nonlinear systems. However, this behavior was intentionally engineered to cater to the erratic stimuli characteristic of wearable and biomedical applications, thereby ensuring a more constant power output once a certain acceleration threshold is exceeded. This design consideration reflects a deliberate trade-off, aiming to accommodate the unpredictable nature of human motion and thus enhance the device’s applicability in real-world scenarios.

The test run at 10 Hz was performed for two assemblies of the EMEH as shown in Figure 8. For low accelerations, a noticeable difference can be observed in performances. This is caused by the spring’s imperfections and slight alignment differences during the assembly. This illustrates that a small change in geometry or mechanical properties can drastically change the behavior of a miniaturized inertial EMEH. Figure 8 shows the results of the proposed inertial EMEH with respect to the state of the art. One can clearly see the critical point at which the externally applied acceleration becomes high enough to trigger the harvester: between [0.5; 0.7] g, the harvester starts to reach other equilibrium positions which results in a higher flux gradient and corresponding power at each coil. Above 0.47 g and 0.69 g respectively, the speed of the inertial proof mass increases and so does the rate of change of the flux in both coils. When the input excitation is sufficiently large (above 0.7 g), one can observe an increase in the power density that boosts the rate at which the core sweeps through the different equilibrium positions. The proposed EMEH produces 848 μW per coil for a total volume of 22.39 cm^3^ under a 1.32 g RMS acceleration. This equals 1696 μW for both coils connected to a resistive load where no PMU losses are considered.

Figure 2 shows that a large range of performances is observed across different designs. Several orders of magnitude in the power density can separate two designs of similar sizes that are submitted to a comparable external excitation. Also, the figure of merit FoMv exhibits this large range of performances, corroborating the observations in previous works [27,31]. Furthermore, the performances drastically decrease when the stimuli do not correspond to the optimum applied frequency and acceleration. Therefore, the performances of the EHs within a broad band of frequencies and accelerations are more relevant than their performances at their maximum yield. Hence, care must be taken to highlight the overall performances, especially for biomedical applications where the movements can be random and sometimes erratic.

Certain limitations warrant attention for further development. Firstly, the device’s size and form factor, while optimized in volume for wearability, present challenges in achieving the ideal balance between physical obtrusiveness and energy harvesting efficiency, a critical aspect for wearable applications where “flat designs” are often preferred to accommodate seamless integration into everyday clothing and accessories without impeding user comfort or aesthetics. Moreover, the longevity and durability of the springs, which require a very low stiffness to facilitate the easy transition between equilibrium positions, merit further investigation. While 3D printing has expedited the prototyping process, enabling swift iterations of our design, it’s evident that a comprehensive mechanical study is essential for developing a durable and resilient system. Unlike our current springs, which are susceptible to deterioration through creep and fatigue, future iterations must focus on materials and manufacturing techniques that prevent such wear over time. This endeavor is crucial to ensure the long-term reliability and effectiveness of the energy harvester in real-world wearable applications. Finally, adapting the Power Management Unit (PMU) to accommodate the unique behaviors and electrical outputs of this energy harvester presents its own set of challenges. The non-linear and variable performance characteristics of our device require a PMU that can efficiently convert and regulate energy across a wide range of conditions. This entails the development of sophisticated algorithms and control mechanisms capable of maximizing energy extraction and storage, regardless of the variability in human movement or environmental conditions.

This 22 cm^3^ size EMEH design can be improved in many ways to fit a given wearable application, such as rehabilitation aids where its size aligns with the device’s structural components, or in portable medical equipment for continuous health monitoring that benefits from the harvester’s substantial energy output. This allows for applications where the device’s volume is not a constraint but rather an asset, such as in knee braces equipped with sensors for gait analysis and improvement, or in smart backpacks designed for real-time health and activity monitoring.

## 6. Conclusions

This paper presents a comparison of the selected state-of-the-art designs where each inertial EMEH was compared on the same footing. A figure of merit based on the idealized generated power for a given volume (FoMv) was introduced to allow a fair comparison. It was observed that less than 10% of power from the idealized power (FoMv) can be generated based on state of the art (with an average at 1.03%).

A dedicated custom test bench was designed to process various EHs operating in a large range of frequencies and accelerations. Experimental validation confirms that the proposed designs exhibit the expected behavior: the equilibrium positions are crossed when the acceleration is sufficient and the two coils spaced by a gap help at reducing the resulting magnetic force and so assist in crossing the equilibrium positions. At 10 Hz and 1.32 g RMS acceleration, the proposed EMEH produces 1696 μW for a total volume of 22.39 cm^3^.

Although the presented analysis with a purely resistive and fixed load facilitates comparison across numerous publications, it is important to note that this does not fully characterize the limitations and performance of the proposed energy harvester under real-world usage conditions. Additional analyses and experimental validations are necessary, particularly focusing on the associated load and the power management unit (PMU) when integrated with the EMEH. Furthermore, the device’s size and wearability and the durability of 3D printed low-stiffness springs require further development to fully meet the stringent demands of wearable and biomedical applications, ensuring that the design not only adheres to but also fully satisfies the specifications outlined in their unique requirements.

## Figures and Tables

**Table 1:** Scenarios used to fix a generic power target for an electromagnetic energy harvester.

Scenario	Sensors	Actuators	Active Time	Mean Power	References
Pacemaker and defibrillator	1 μW	1 W	Always	28.5 μW	[12]
Gait cycle monitoring	10 mW	-	When moving	30 mW	[13]
Neurostimulator	120 μW	1 mW	Always	60 μW	[14,15]

**Table 2:** List of parameters and dimensions of the proposed and tested EMEH.

Parameter	Value/Range	Unit	Comment
$V_{matter}$	10.90	cm^3^	
$V_{tot}$	22.39	cm^3^	Total volume
$m_{core}$	10.26	g	Fix parts (wounded cores, Winding and brass axis)
$m_{frame}$	61.70	g	Proof-mass (magnets and brass frame)
$m_{spring}$	2.37	g	
$m_{Tot}$	74.33	g	Total mass
*L*	18	mm	Height of the harvester
*l*	2	mm	
*R*	16.5	mm	Outer radius of the harvester
*r*	4.5	mm	Radius of the coil
*G*	0.05	mm	Gap between magnet and core
*g*	1.83	mm	Gap between both coils
$N_{wire}$	800	-	Number of turns for one coil
$R_{wire}$	23	Ω	Resistance of the coil
$L_{coil}$	12	mH	Inductance of the coil
$R_{Load}$	25	Ω	Load resistors connected to coils

## Data Availability

Data are contained within the article.

## Abbreviations

The following abbreviations are used in this manuscript:

EH	Energy Harvester
EMEH	Electromagnetic Energy Harvester
FEA	Finite Element Analysis
FoMv	Volume-based Figure of Merit
PMU	Power Management Unit
